# Cutaneous Deficiency of Filaggrin and STAT3 Exacerbates Vaccinia Disease *In Vivo*

**DOI:** 10.1371/journal.pone.0170070

**Published:** 2017-01-12

**Authors:** Yong He, Ishrat Sultana, Kazuyo Takeda, Jennifer L. Reed

**Affiliations:** Food and Drug Administration, Center for Biologics Evaluation and Research, 10903 New Hampshire Ave., Silver Spring, MD, United States of America; NYU Langone Medical Center, UNITED STATES

## Abstract

**Rationale:**

Defects in filaggrin and STAT3 are associated with atopic dermatitis (AD) and susceptibility to severe skin infection.

**Methods:**

We evaluated skin infection with the current smallpox vaccine, ACAM-2000, in immunosuppressed mice with combined cutaneous deficiency in filaggrin and STAT3. In parallel, early events post-infection with ACAM-2000 were investigated in cultured keratinocytes in which filaggrin expression was knocked down via siRNA.

**Results:**

Immunosuppressed, filaggrin-deficient mice, treated with the topical STAT3 inhibitor Stattic® prior to ACAM-2000 infection, demonstrated rapid weight loss, prolonged vaccinia burden in skin, and dermatitis. The TGF-β family ligand activin A was upregulated ten-fold in infected skin. Topically-applied ALK5/TGβR1 signaling inhibitor synergized with vaccinia immune globulin (VIG) to promote vaccinia clearance and limit weight loss. In cultured keratinocytes, filaggrin-directed siRNA inhibited programmed necrosis and inflammatory cytokine release induced by ACAM-2000, while viral growth was increased.

**Conclusions:**

Our findings may point to a novel role for filaggrin in early antiviral responses in skin. In wounded skin with underlying barrier defects, chronically elevated activin A levels may contribute to skin remodeling and cutaneous pathogen persistence. Inhibition of ALK5/TGFβR1 signaling may provide a novel co-therapeutic approach, together with VIG, to limit cutaneous spread of vaccinia.

## Introduction

Vaccinia virus, a dermotropic member of the orthopox family, is the active component of the smallpox vaccine. Vaccinia scarification usually results in a skin lesion which ulcerates and gradually heals over a period of several weeks, coinciding with the development of antiviral cell mediated and humoral immunity. Recent studies have begun to link innate responses of skin with limiting vaccinia spread and contributing to successful vaccination outcome [1]. Correspondingly, defects in genes regulating cutaneous barrier function have been implicated in eczema vaccinatum (EV), the catastrophic skin infection that occurs when individuals with atopic dermatitis (AD) or other skin disorders are accidentally exposed to vaccinia [2]. The precise anti-vaccinia contributions of the cutaneous barrier remain an area of active study.

Dominant negative mutation of the STAT3 gene is one innate defect associated with AD and severe skin infection susceptibility from infancy [3]. Previously, we used the licensed smallpox vaccine, ACAM-2000, in a severe combined immunodeficient (SCID) mouse model to evaluate possible anti-vaccinial contributions of STAT3 [4]. Vaccinia-scarified SCID mice, treated topically prior to infection with an inhibitor of phosphorylated and non-phosphorylated STAT3 (Stattic®), demonstrated larger vaccinia lesions, higher viral titers, and shorter survival post-infection, compared to scarified, vehicle control-treated animals. In addition, inhibition of STAT3 in cultured keratinocytes significantly increased virus recovery, and reduced antiviral responses to vaccinia infection such as rapid necrosis of infected cells, activation of type I interferon, and inflammatory cytokine release [4]. Together these data suggested thatSTAT3-dependent innate responses in the skin might critically limit viral spread early after exposure. Because skin barrier integrity is a polygenic trait [5], we considered whether combinations of gene defects linked with barrier dysfunction might synergize to further increase vaccinia susceptibility.

In the past decade, the multifunctional corneocyte protein filaggrin has emerged as essential for cutaneous barrier function, with various loss-of-function mutations associated with lifelong susceptibility to severe skin infection [5–7]. In the current study, we modeled for the first time ACAM-2000 infection in immunosuppressed mice with deficiency in two skin barrier genes. Immunosuppressed mice with cutaneous deficiency in both STAT3 and filaggrin displayed rapidly progressing vaccinia disease, characterized by elevated local virus recovery, dermatitis, mucosal mast cell accumulation, and activin A overexpression in infected skin. Post-exposure blockade of activin A signaling with a topically-applied ALK5/ TGFβR1 inhibitor synergized with vaccinia immune globulin (VIG) to limit disease and reduce virus recovery. In vitro, filaggrin-directed siRNA increased viral growth in ACAM-2000 infected human keratinocytes, while early antiviral responses were reduced. These data are the first to our knowledge to implicate infection-induced TGFβR family signaling in cutaneous persistence of vaccinia. The data may suggest new roles for filaggrin in pathogen sensing in the skin, and could support additional keratinocyte-based screening for novel host factors and pathways essential for cutaneous antiviral defense.

## Materials and Methods

### Mice

All experiments were approved by the Intramural Animal Care and Use Committee of the Center for Biologics Evaluation and Research, Food and Drug Administration and carried out in strict adherence to protocol, including efforts to minimize suffering of study animals. Mice were housed and maintained according to NIH Animal Research Advisory Committee guidelines. Six to 12 week old SCID/NCr mice were obtained from the NCI Frederick Animal Production Program. Filaggrin-deficient “flaky tail” (FT) mice were purchased from Jackson Laboratories. Animals on study were monitored daily for signs of illness. Any animals, whether on study or not, that exhibited a moribund or hunched appearance, ruffled fur, inability to reach food or water, or that weighed less than 80% starting weight, were promptly euthanized by CO2 inhalation according to the IACUC-approved protocol. Study animals received Nutrigel nutritional support and acetaminophen in drinking water to relieve distress. In the studies described, no animals died prior to expected experimental endpoints.

### Vaccinia virus strains and stock preparation

VACV-ACAM-2000 (Acambis, Inc., Cambridge, MA), a vaccine strain clonally derived from Dryvax®, was obtained through the Centers for Disease Control. Virus stock was prepared in Vero E6 cells (ATCC, Manassas, VA) and stored as previously described [4].

### In Vivo STAT3 Inhibition and Scarification

The STAT3 inhibitor Stattic® was prepared and applied prior to vaccinia scarification as previously described [4]. Cyclophosphamide (Santa Cruz Biotech, Dallas, TX) was administered by intraperitoneal route starting with the day prior to vaccinia scarification and every 4 days after vaccinia infection as previously described [8]. Scarification using 10^6^ pfu ACAM-2000 was performed as previously described [4].

### Plaque assay

Skin biopsy tissues collected from euthanized mice were weighed and homogenized in cDMEM. Quantitation of recovered virus in homogenates was performed by plaque assay on Vero E6 cells as previously described [4].

### ELISA

Monoplex ELISA kits to detect murine activin A, human activin A, and human cytokines were purchased from RandD Systems (Minneapolis, MN) and used according to manufacturer’s instructions. Proinflammatory cytokines were measured in trioxsalen/UV-inactivated vaccinia infected cell culture supernatant, as previously described [4].

### Immunohistochemistry and immunoblot

Primary antibodies reactive with mouse Sca-1, STAT3, TAK1, phospho-TAK1 (Thr184/187), phospho-STAT3 (Tyr705), and β-actin (Cell Signaling Technology, Danvers, MA), murine mast cell protease-1 (mMCPT1; BioLegend, San Diego, CA), filaggrin (Abcam, Cambridge, MA) and vaccinia antigens (Santa Cruz Biotech, Dallas, TX) were used according to manufacturer recommendations. Formalin-fixed, paraffin-embedded skin tissues were sectioned and processed as previously described [4]. Cell cytosolic fractionation and immunoblot of HEK-001 cell lysates were analyzed as previously described [4].

### Cell viability assays

Cell viability was assessed as previously described [4] using a Cell Titer-Glo Luminescent Cell Viability Assay Kit (Promega, Madison, WI) according to manufacturer’s instructions. Values are presented as percent of uninfected vehicle control cells assessed in parallel with test samples.

### Real time PCR

HEK-001 keratinocytes were collected in TRIzol reagent (QIAGEN, Valencia, CA). cDNA was synthesized from total RNA using a quantiTect Reverse Transcription Kit (QIAGEN, Valencia, CA). SYBR Green Premix was used for quantitative PCR with a CFX96 Touch Real-Time PCR System (BioRad., Hercules, CA). Primers used for filaggrin detection: forward 5’ GCTGAAGGAACTTCTGGAAAAG3’, reverse 5’GCCAACTTGAATACCATCAGAAG3’. Primers for detection of β-actin (control): forward 5’ GTCTTCCCCTCCATCGTG3’, reverse 5’ GTACTTCAGGGTGAGGATGC3’.

### Transient RNA interference and transfections

Transcripts were targeted in keratinocytes using a pool of small short interfering RNAs (ON-TARGET Plus Smart Pool, Thermo, CO, USA) as previously described [4].

### Statistical analysis

Statistical analysis was performed using Prism 5 software (GraphPad Software, La Jolla, CA). Differences between groups were assessed by t-test, with statistical significance defined as p≤0.05.

## Results

### Mice with filaggrin deficiency and STAT3 inhibition in skin display severe disease and involvement of distant skin after ACAM-2000 scarification

**Fig 1:**
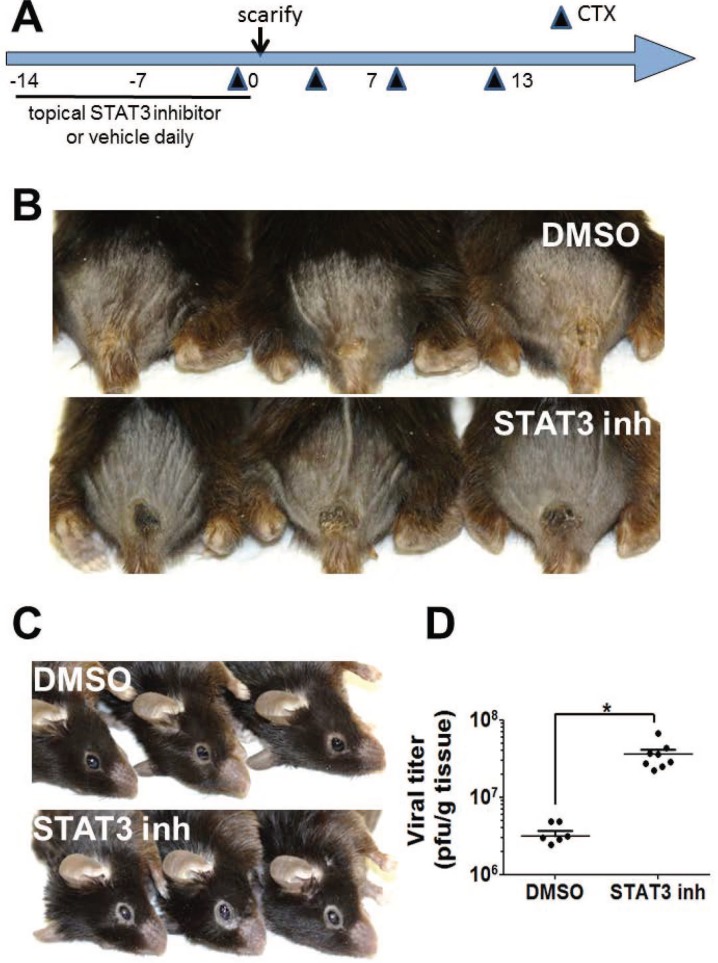
Topical STAT3 inhibition is associated with increased viral recovery in immunosuppressed, filaggrin deficient mice. A) Schematic protocol. DMSO vehicle or the STAT3 specific inhibitor Stattic® was applied topically, for two weeks before ACAM-2000 scarification in cyclophosphamide (CTX) immunosuppressed FT mice. Representative vaccinia lesions (B) and beginning dermatitis of face (C) in mice pretreated with topical DMSO (upper row) or Stattic® (lower row) before ACAM-2000 scarification, photographed on day 13. (D) Virus recovery on day 13 was assessed in homogenized primary lesion tissue (n = 9–10). Asterisk, p≤0.01.

A spontaneous frameshift mutation in the murine filaggrin gene has been linked with cutaneous barrier deficit, robust percutaneous allergen priming, and enhanced inflammatory responses to topically applied haptens in “flaky tail” (FT) mice [9–10]. Studies of ACAM-2000 infection in FT mice have not been previously published. To extend our evaluation of anti-vaccinia to FT mice, which are euthymic, we adapted a published vaccinia scarification model in immunosuppressed hairless mice [8] (Fig 1). Briefly, FT mice pretreated with the immunosuppressant cyclophosphamide (Cytoxan, CTX) and topical STAT3 inhibitor or vehicle were scarified with ACAM-2000 (10^6^ pfu), applied with a bifurcated needle in two locations 2 mm apart on the shaved back of mice. In immunosuppressed FT mice, ACAM-2000 scarification produced a small, flat lesion at 13 days post-inoculation (Fig 1). On the other hand, immunosuppressed FT mice, pretreated with topical Stattic® before ACAM-2000 scarification, had larger primary lesions with hair regrowth within the lesion boundary, with evidence of scratching of eyes and muzzle by day 13 (Fig 1). These features were not observed in uninfected mice, nor in infected mice that received topical DMSO treatment prior to ACAM-2000 infection. Local vaccinia recovery on day 13 was significantly higher in FT mice that had received topical STAT3 inhibitor (Fig 1), supporting the possibility that excess virus growth might be causally related to the altered lesion appearance and scratching behavior.

**Fig 2:**
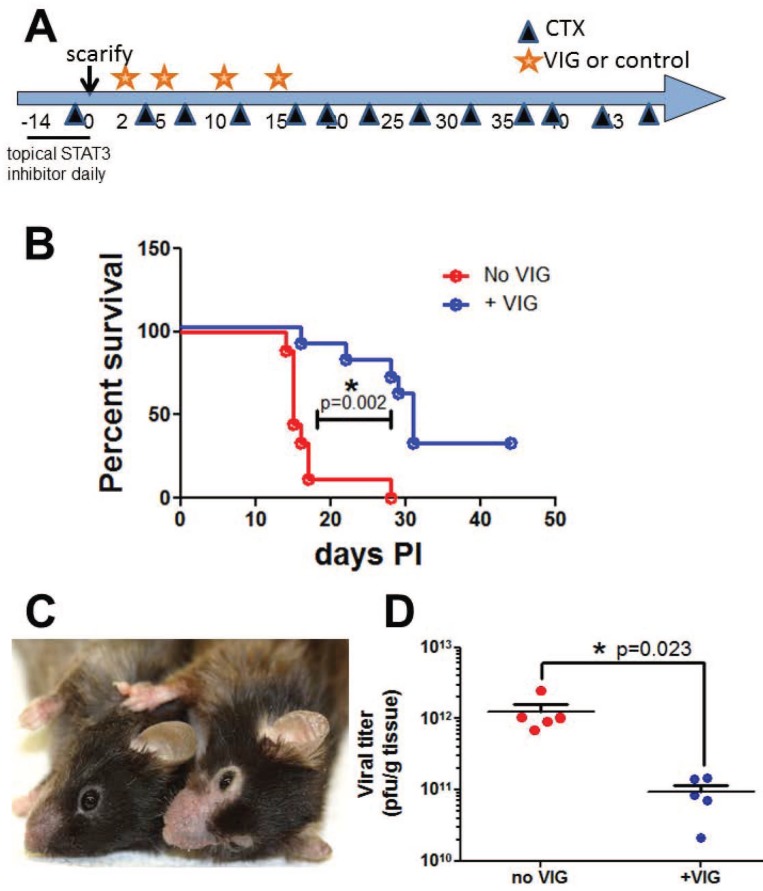
Topical STAT3 inhibition is associated with severe vaccinia disease and dermatitis in CTX treated FT mice. A) Schematic protocol. The STAT3 specific inhibitor Stattic® was applied topically for two weeks before ACAM-2000 scarification in CTX treated FT mice. Animals received VIG or vehicle control on days 2, 5, 10 and 15. B) Rapidly progressing vaccinia disease was significantly delayed by VIG (n = 9–10). C) Representative uninfected (left) and infected (right) mice. D) Virus recovery from primary lesions on day 20 (n = 5 per group). Viral burden in skin was significantly reduced in VIG recipients. Representative data from 2 experiments are shown.

Follow-up studies evaluated whether post-exposure VIG, administered using a published protocol, had an ameliorative effect on vaccinia pathogenesis in immunosuppressed, Stattic®-pretreated FT mice [4, 11] (Fig 2). Scarified mice that received mock therapeutic treatment rapidly lost weight, with 90% of the group reaching predefined euthanasia criteria by day 20. Mice receiving post-exposure VIG lived longer, but VIG was less protective in infected FT mice than had been previously observed in the SCID model [11] (Fig 2). VIG treatment reduced virus recovery in the primary lesion. Dermatitis of ears, paws, and muzzle was observed in infected animals of both groups (Fig 2).

**Fig 3:**
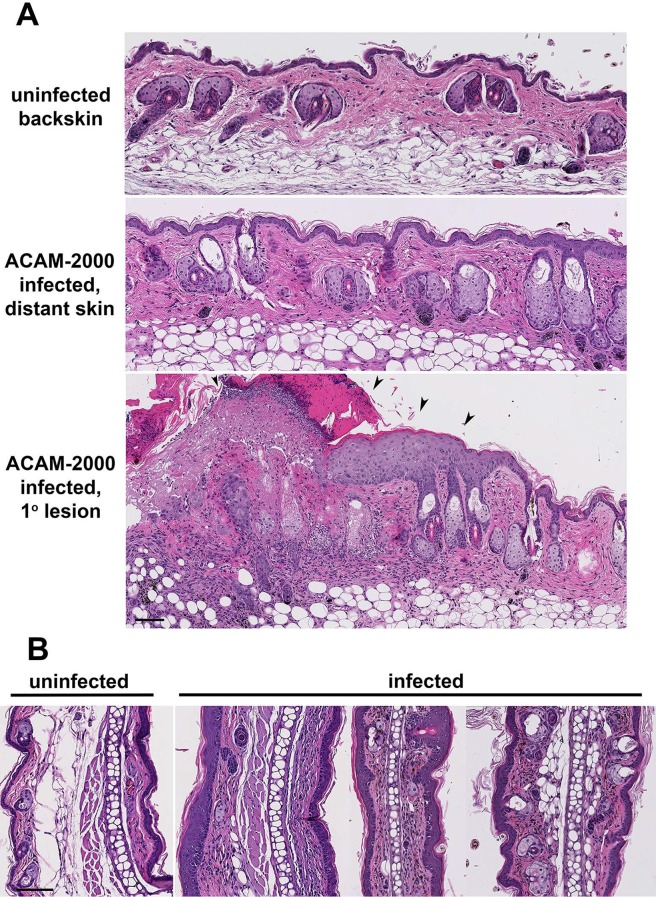
Histological evaluation distant skin of infected FT mice. The STAT3 specific inhibitor Stattic® was applied topically for two weeks before ACAM2000 scarification in CTX treated FT mice. On day 20, back skin (A; bar = 500 μm) and ear tissue (B; bar = 250 μm) was collected from uninfected infected mice. Formalin-fixed paraffin embedded tissue was sectioned for H+E staining and histological analysis. Arrowheads: vaccinia lesion. Representative of 3 experiments is shown.

We performed histologic evaluation of tissues from ACAM-2000 infected FT mice to identify potential mechanisms of increased disease severity in this model. Uninfected back skin of FT mice appeared histologically normal (Fig 3A). At day 20 post-ACAM-2000 scarification, increased cellularity and collagen deposition was observed in primary lesion skin, in back skin distant from the primary lesion, and in ear tissue (Fig 3). However viral antigens were only detected in the primary lesion (S1 Fig and data not shown). Stem cell antigen-1 (Sca-1) positive cells were numerous in the epidermis, follicles and sebaceous glands of the primary lesion (S1 Fig). Toluidine blue-positive mast cells accumulated in granulation tissue of the primary lesion were unexpectedly positive for murine mast cell protease-1 (mMCP-1), a chymase that in rodents is highly specific to mast cells of mucosal tissue, not skin mast cells (S2 Fig).

### Evidence for activin A contribution to severe vaccinia disease in FT mice

**Fig 4:**
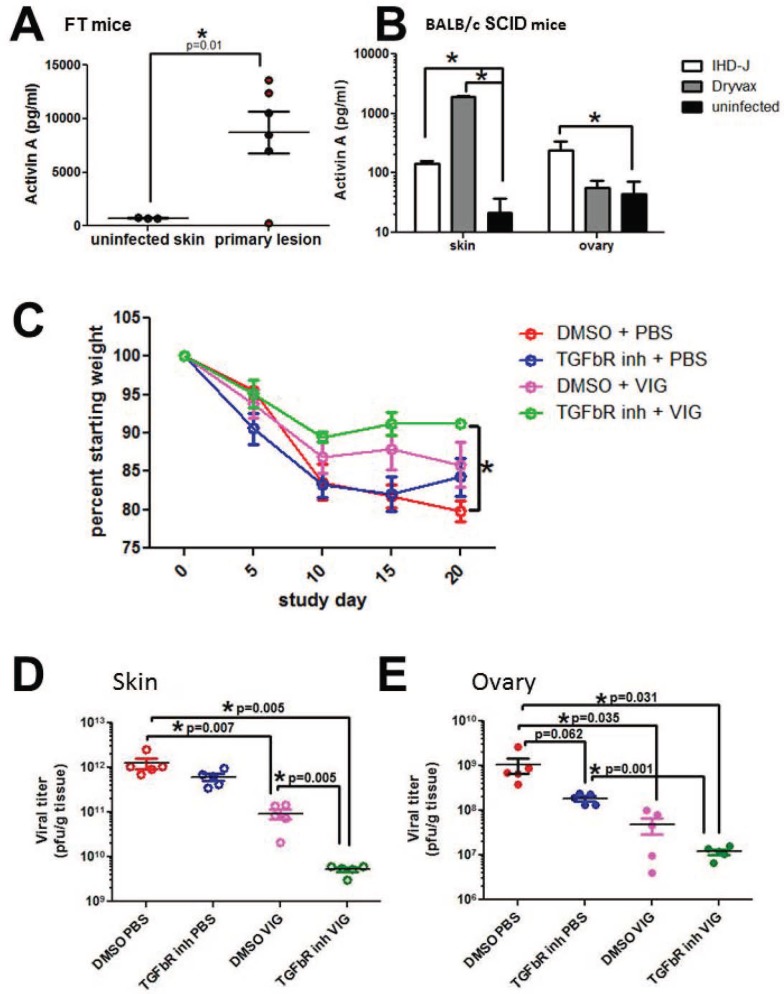
Activin A in vaccinia infection of mice. Activin A was detected by ELISA in tissue homogenates of primary vaccinia lesions from (A) flaky tail mice infected with ACAM-2000, and (B) retain lesional tissue from SCID mice infected with vaccinia strains IHDJ and Dryvax. (C-E) ACAM-2000 infection was carried out in CTX treated FT mice as described in Fig 2. The TGFβR inhibitor SB431542, or vehicle control, was applied topically beginning on day 1 post infection. On day 20, primary lesion skin (D) and ovary tissue (E) were collected and viral burden was analyzed by plaque assay. Representative data from 2 experiments are shown. Asterisk, p≤0.05, or as noted in figure.

Induction of mMCP-1 expression in mast cells is associated with specific stimuli, such as IL-9 and activin A [12–13]. ELISA evaluation of tissue homogenates from primary vaccinia lesions demonstrated activin A protein (but not IL-9, not shown) in primary ACAM-2000 lesions of FT mice (Fig 4). An evaluation of retain samples from BALB/c SCID mouse studies also demonstrated activin A in vaccinia lesions, but at lower levels than found in infected FT mice. We considered whether increased expression of activin A, a TGF-β family ligand linked with cachexia in people and mice [14–15], might be contributing to weight loss observed in mice infected with vaccinia virus. To test this hypothesis, primary ACAM-2000 lesions of FT mice were topically treated with SB431542, a specific inhibitor of TGF-β superfamily type I receptor kinases ALK4, ALK5, and ALK7 that can transduce activin A signals [15] (Fig 4). Topically-applied SB431542 synergized with VIG to stabilize weight and reduce viral recovery from skin and ovary in infected FT mice, while SB341542 alone had no significant effect. These results could suggest that activin A overexpression in FT mice may contribute to exaggerated disease phenotype observed with ACAM-2000 scarification.

### Potential STAT3 and filaggrin antiviral contributions in vitro: studies in cultured keratinocytes

**Fig 5:**
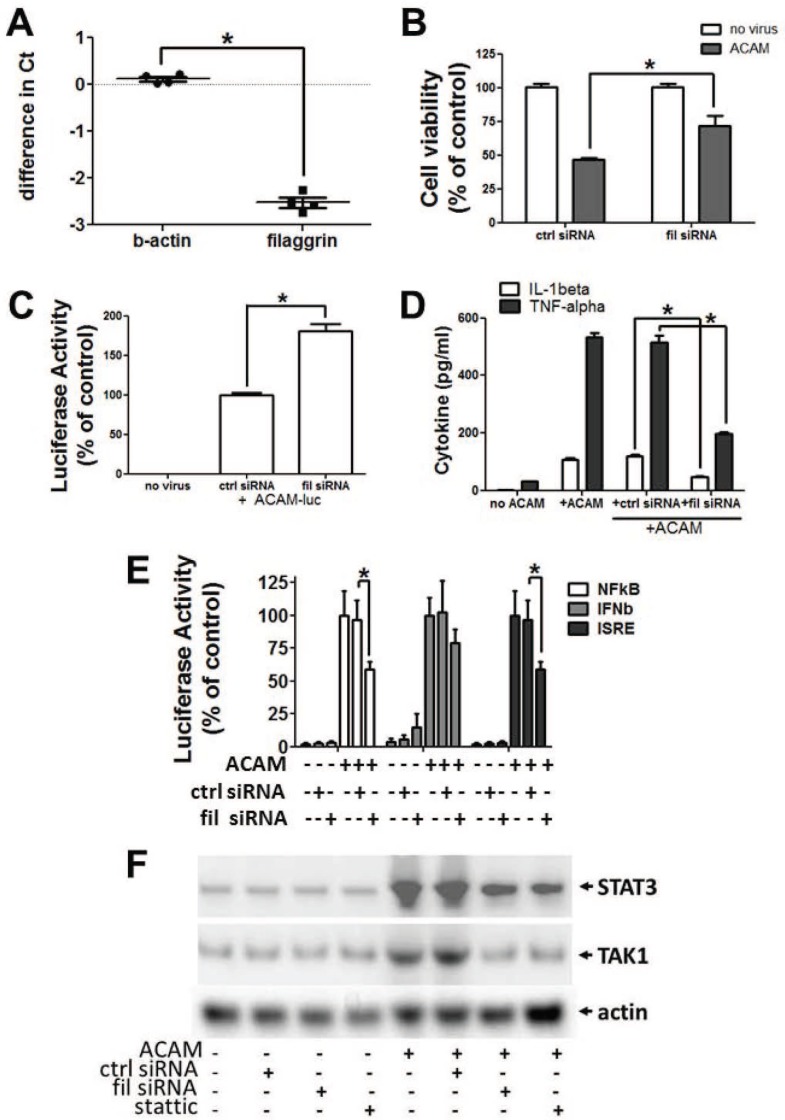
Filaggrin in antiviral responses of cultured human keratinocytes. Human keratinocyte HEK-001 cells were transfected with control or filaggrin-targeted siRNA, 20 nM concentration, 48 hours prior to tests. A) Abundance of β-actin and filaggrin mRNA is expressed as a ratio of test siRNA: control siRNA wells (n = 4). B) Cells were infected with ACAM2000 at 20 MOI or mock challenged. Viability at 12 hours post challenge (n = 4) was evaluated by ATP release. (C) Cells were infected with recombinant ACAM-luc at 1 MOI. Cells were harvested at 12 hours for detection of viral luciferase. D) Cytokines were measured in culture supernatant 48 hours post infection with ACAM-2000 at 20 MOI. E) Cells were co- transfected with siRNA and reporter plasmids encoding luciferase downstream of NFkB, IFNb, or ISRE promoter elements. After 48 hours, transfected cells were infected with ACAM-2000 at 20 MOI. Luciferase signal was measured at 24 hours postinfection. F) HEK-001 were infected with ACAM-2000 at 20 MOI. Hypotonic lysates collected at 3 hours post-infection were analyzed by immunoblot. Representative of at least 3 experiments is shown. Asterisk, p≤0.05.

For additional mechanistic insight into potential anti-vaccinia roles for filaggrin, we used an ACAM-2000 in vitro infection system previously established in human HEK-001 keratinocytes [4]. Expression of filaggrin was confirmed in HEK-001 by immunoblotting with a filaggrin-directed antibody (S3 Fig), and by quantitative RT-PCR (Fig 5). Commercially available siRNA reduced filaggrin transcript in HEK-001 cells by 82% as calculated by ΔΔCT method in replicate wells (Fig 5). Filaggrin-directed siRNA, applied prior to ACAM-2000 challenge at 20 MOI, reduced rapid death of infected keratinocytes, while scrambled control siRNA had no effect (Fig 5). To evaluate vaccinia growth in the context of filaggrin knockdown, we used a previously described recombinant, luciferase-expressing strain of ACAM-2000 [4]. Vaccinia-associated luciferase signal was significantly increased in keratinocytes treated with filaggrin-directed siRNA prior to infection, compared with keratinocytes identically pretreated with scrambled control siRNA (Fig 5), possibly implicating filaggrin in rapid necrosis of infected cells.

Because programmed necrosis is a proinflammatory process, we evaluated whether filaggrin-directed siRNA might also reduce inflammatory signaling and cytokine responses in ACAM-2000 infected keratinocytes. Prior to ACAM-2000 infection, keratinocytes were co-transfected with siRNA plus reporter plasmids directing luciferase expression under the control of NF-κB, IFN-β, or IRF3 promoter elements. Increased NF-κB, IFN-β, and IRF3 promoter activity was observed after vaccinia infection (Fig 5), and pretreatment with filaggrin-inhibitory siRNA, but not control siRNA, significantly reduced luciferase reporter activity directed by NF-kB and IRF3 promoters, but not IFN-β. Pretreatment with filaggrin-specific siRNA, but not control siRNA, also limited the release of proinflammatory cytokines TNF-α and IL-1β from ACAM-2000 infected keratinocytes (Fig 5), while there was no impact on activin A release from infected cells (S4 Fig). Since the effects of filaggrin-directed siRNA appeared congruent with effects of STAT3 inhibition in the same infection system, we evaluated whether filaggrin might intersect with STAT3 signaling in infected keratinocytes. Pretreatment of keratinocytes with filaggrin-specific siRNA, but not control siRNA, inhibited the accumulation of both STAT3 and the innate immune signaling kinase TAK1 in the cytosol 3 hours after high MOI vaccinia infection (Fig 5). Similarly, phosphorylated forms of STAT3 and TAK1, detected in keratinocyte cytosol 3 hours post-infection, were reduced by filaggrin-specific siRNA but not control siRNA (S5 Fig). These effects appeared similar to that of the small-molecule STAT3 inhibitor Stattic®.

## Discussion

These studies aimed to evaluate contributions of the skin toward successful anti-vaccinia responses after scarification. The data address for the first time vaccinia infection in a model featuring combined cutaneous deficiencies in proteins associated with skin barrier dysfunction. The data provide evidence of synergy between cutaneous inhibition of STAT3 and constitutive filaggrin deficiency, allowing increased ACAM-2000 replication in skin and enhanced vaccinia disease. Dermatitis and skin remodeling observed in this model have not been reported in previous murine models of vaccinia infection, not observed in infected mice with filaggrin deficiency alone, and not found in infected mice with normal filaggrin expression that received topical STAT3 inhibitor treatment [4]. These observations could support a polygenic model of skin barrier function, in which combinations of polymorphisms critically regulate skin infection susceptibility and disease outcomes. Together, the data provide further evidence that early responses of keratinocytes restrict vaccinia replication and shape the course of infection.

How filaggrin impacts keratinocyte responses to vaccinia infection is still under investigation. Previous studies have shown filaggrin interacts with keratin intermediate filaments, forming a scaffold for lipids of the insoluble cornified envelope. In addition, degradation products of filaggrin are major constituents of hygroscopic natural moisturizing factor (NMF)[16– 17]. Insufficient lipid and NMF in patients with filaggrin deficiency has been proposed underlie barrier integrity loss (exacerbated by scratching), increase antigen presentation, exaggerate Th2- and Th17 responses, and reduced antimicrobial peptide expression [16]. The current in vitro data may point to a different role for filaggrin in pathogen sensing and innate signaling in infected keratinocytes. Filaggrin possibly could exert this effect through regulation of keratin intermediate filaments, which in other cell systems have important effects on cell death pathways triggered by inflammatory stimuli [18]. One possibility is that filaggrin interaction with intermediate filaments helps to pre-assemble necroptotic machinery in keratinocytes, as was recently suggested in macrophages [19], permitting rapid response to innate triggers. These and other possible contributions of filaggrin to innate immune signaling in keratinocytes are the focus of ongoing work.

Activin A is strongly induced in wounded skin, and activin A accelerates wound resolution without mast cell requirement in mice with a normal epidermal barrier [20–21]. The current data are the first to our knowledge to illustrate the potential for untoward effects of activin A overexpression, when underlying barrier defects delay or preclude wound resolution. In skin with barrier defects, chronic overexpression of activin A may expand the population of pluripotential cells. Although responses of semi-differentiated cells to vaccinia infection have not been previously studied, less differentiated cells may have reduced innate responses and might potentially contribute to a viral replicative niche in the skin [22]. Mucosally-skewed mast cells, recruited and activated by epidermally-derived activin A, are a potential source of anti-inflammatory and pro-fibrotic mediators which may also critically support a viral niche. While anti-vaccinia functions for degranulation products of mast cells have been reported, the data were collected using mouse models featuring intact skin barrier and low, self-limiting vaccinia infection of the skin [23]. Our future studies will determine whether targeting mast cell-dependent skin remodeling has potential utility for controlling cutaneous vaccinia in susceptible patients.

Although naturally-occurring smallpox is no longer a threat, the possibility of variola use as a bioterrorism weapon prompts continued vaccination of military personnel and first responders with ACAM-2000 [2]. Because ACAM-2000 effectively elicits T-lymphocyte responses and poxvirus- neutralizing antibodies, the vaccine remains in the national stockpile should events require expansion of the vaccination program. However, wider exposure of the public to ACAM-2000 would be expected to result in hundreds or thousands of cases of disseminated vaccinia [2]. The current data set provides new insights into how defects in cutaneous innate responses in susceptible individuals may drive viral persistence, in part through excessive elaboration of wound healing factors. Further analysis of the intersection between cutaneous pathogen sensing and wound healing may help prepare for a future emergency, by identifying dysregulated anti-pathogen responses in susceptible individuals, and providing new, VIG-sparing opportunities for their treatment.

## Supporting Information

S1 Fig Immunohistochemical Analysis of Vaccinia Lesion Tissue from ACAM-2000 Scarified FT Mice, Day 20 Post-Infection. STAT3 specific inhibitor was applied topically for two weeks before ACAM-2000 scarification in CTX- immunosuppressed, filaggrin deficient mice. Primary lesions were collected on day 20 for histological analysis. Vaccinia antigen detection (top) and Sca-1 antigen (center) localized to keratinocytes of the epidermis, follicles, and sebaceous glands. Bottom: no primary antibody negative control. Representative of 3 experiments is shown. Bar = 100μm.
(TIF)

S2 Fig Immunohistochemical and Immunofluorescence Analysis of Vaccinia Lesion Tissue from ACAM-2000 Scarified FT Mice, Day 20 Post-Infection. STAT3 specific inhibitor was applied topically for two weeks before ACAM-2000 scarification in CTX- immunosuppressed, filaggrin deficient mice. Primary lesions were collected on day 20 for histological analysis. Top: toluidine blue staining identifies mast cells (purple) in granulation tissue. Bottom: murine mast cell protease-1 (mMCP-1, green) and vaccinia antigen (red) detection in primary lesion. Representative of 3 experiments is shown. Bar = 100μm.
(TIF)

S3 Fig Detection of Mouse and Human Filaggrin by Immunoblot. Tail skin samples from mice of BALB/c (lane 1), C57Bl/6 (lanes 2–3), and flaky tail (lanes 4–5) were homogenized in sample buffer. HEK-001 cells that were mock-transfected (lane 6), or transfected with filaggrin-directed siRNA (lane 7) or scrambled control siRNA (lane 8) for 48 hours were collected and extracted in whole cell lysis buffer. Samples were analyzed by SDS-PAGE and immunoblot using anti-filaggrin polyclonal antibody generated in rabbit. Arrow: 28 kDa, the predicted size of monomeric filaggrin.
(TIF)

S4 Fig Secretion of Activin A by Vaccinia-Infected Cultured Human Keratinocytes. HEK-001 were mock-infected (white bar) or infected with ACAM-2000 at 20 MOI (grey bar). Activin A protein was assessed in supernatants collected at 48 hours post infection using a commercially available monoplex ELISA kit (n = 4). Small differences between groups were not statistically significant.
(TIF)

S5 Fig Filaggrin-Dependent Detection of Phosphorylated STAT3 and Phosphorylated TAK1 in Keratinocyte Cytosol 3 Hours Post-Infection. HEK-001 were infected with ACAM-2000 at 20 MOI. Hypotonic lysates collected at 3 hours post-infection were analyzed by immunoblot. Representative of 2 experiments is shown.
(TIF)
