# Supplementary figures and images for: Cutaneous Deficiency of Filaggrin and STAT3 Exacerbates Vaccinia Disease In Vivo
Source: PLoS One. 2017 Jan 12;12(1):e0170070. doi: 10.1371/journal.pone.0170070 (PMC5231274; doi:10.1371/journal.pone.0170070)

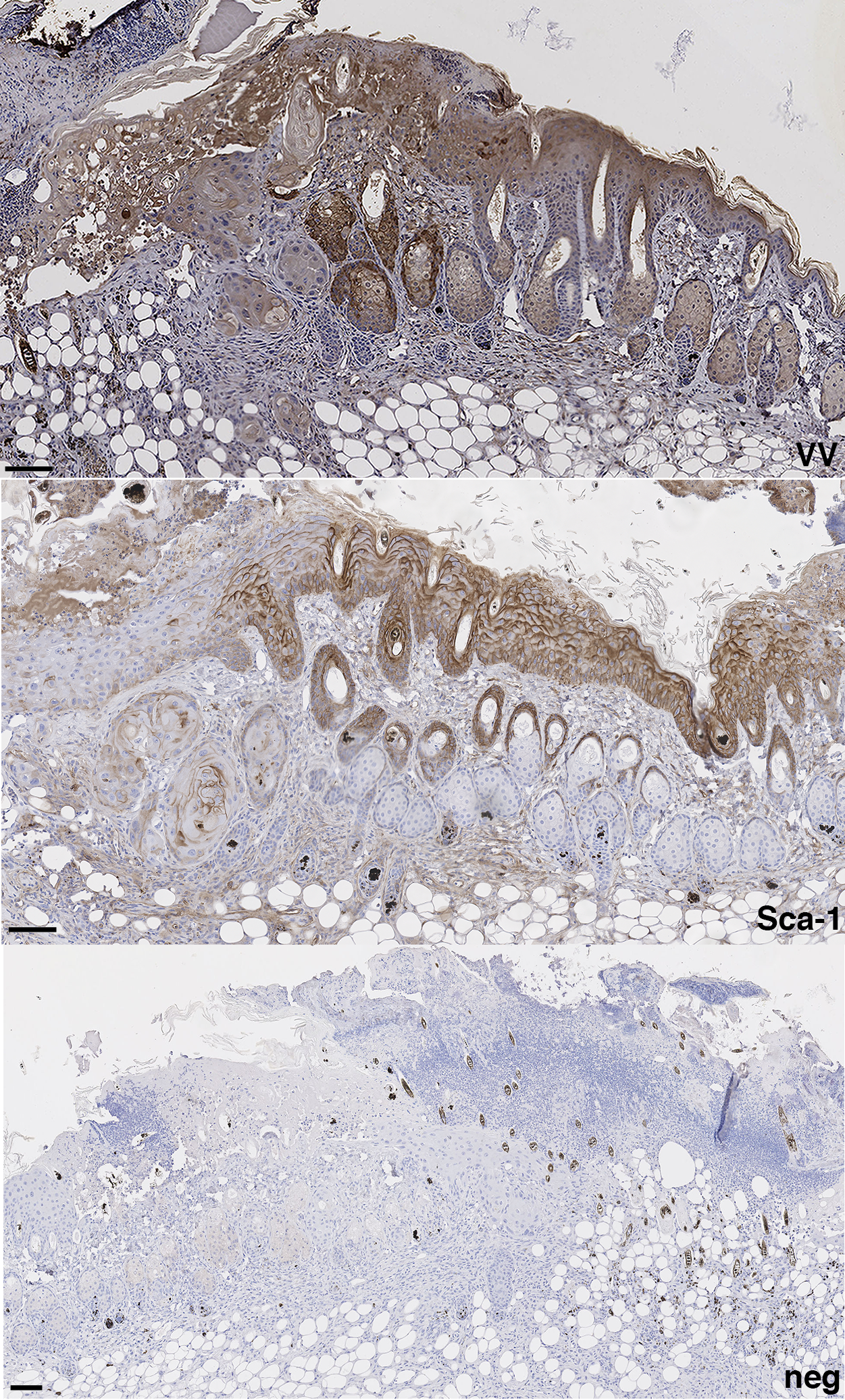

Supplement: S1 Fig — STAT3 specific inhibitor was applied topically for two weeks before ACAM-2000 scarification in CTX- immunosuppressed, filaggrin deficient mice. Primary lesions were collected on day 20 for histological analysis. Vaccinia antigen detection (top) and Sca-1 antigen (center) localized to keratinocytes of the epidermis, follicles, and sebaceous glands. Bottom: no primary antibody negative control. Representative of 3 experiments is shown. Bar = 100μm. (TIF) [file pone.0170070.s001.tif]

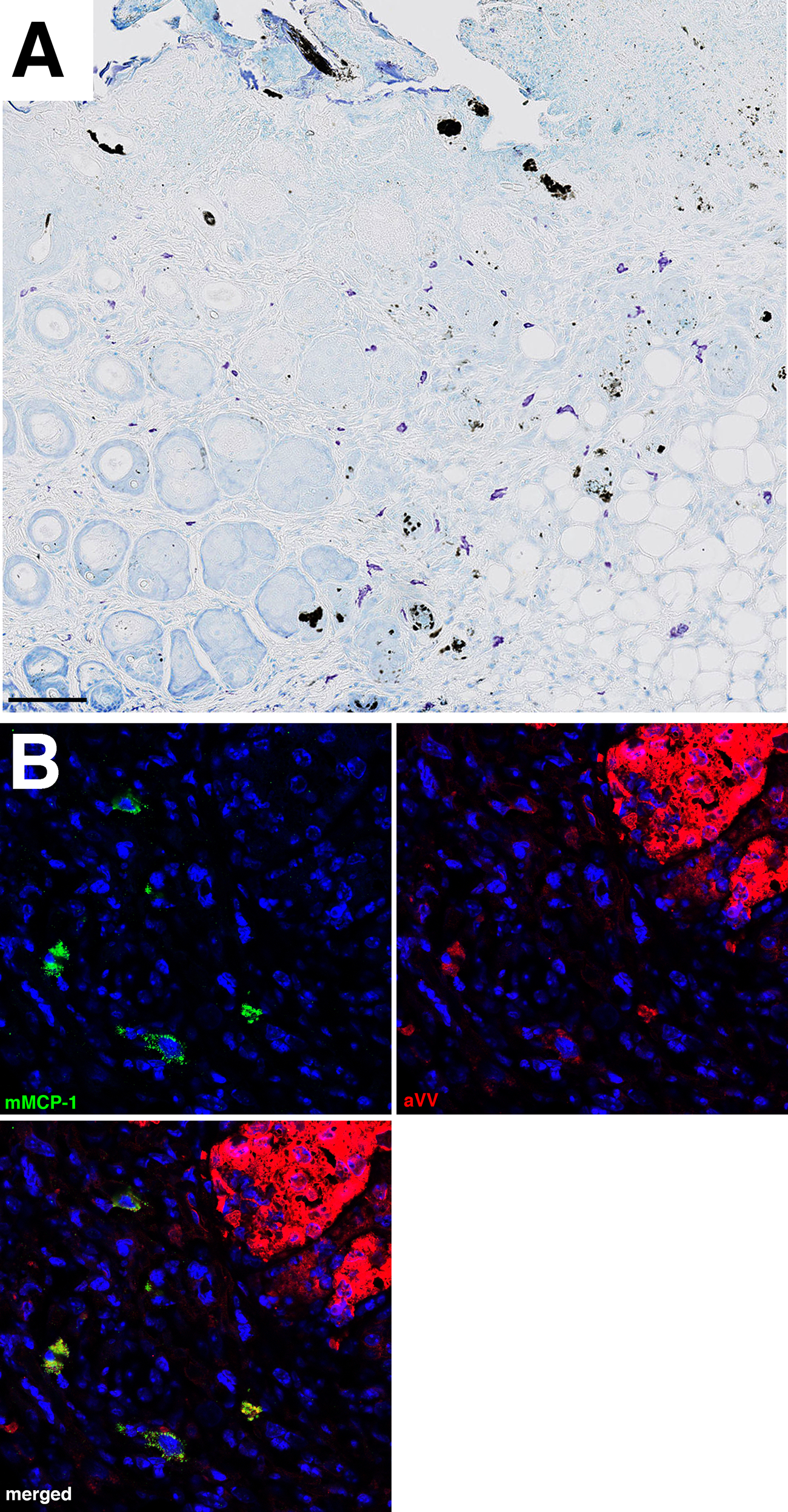

Supplement: S2 Fig — STAT3 specific inhibitor was applied topically for two weeks before ACAM-2000 scarification in CTX- immunosuppressed, filaggrin deficient mice. Primary lesions were collected on day 20 for histological analysis. Top: toluidine blue staining identifies mast cells (purple) in granulation tissue. Bottom: murine mast cell protease-1 (mMCP-1, green) and vaccinia antigen (red) detection in primary lesion. Representative of 3 experiments is shown. Bar = 100μm. (TIF) [file pone.0170070.s002.tif]

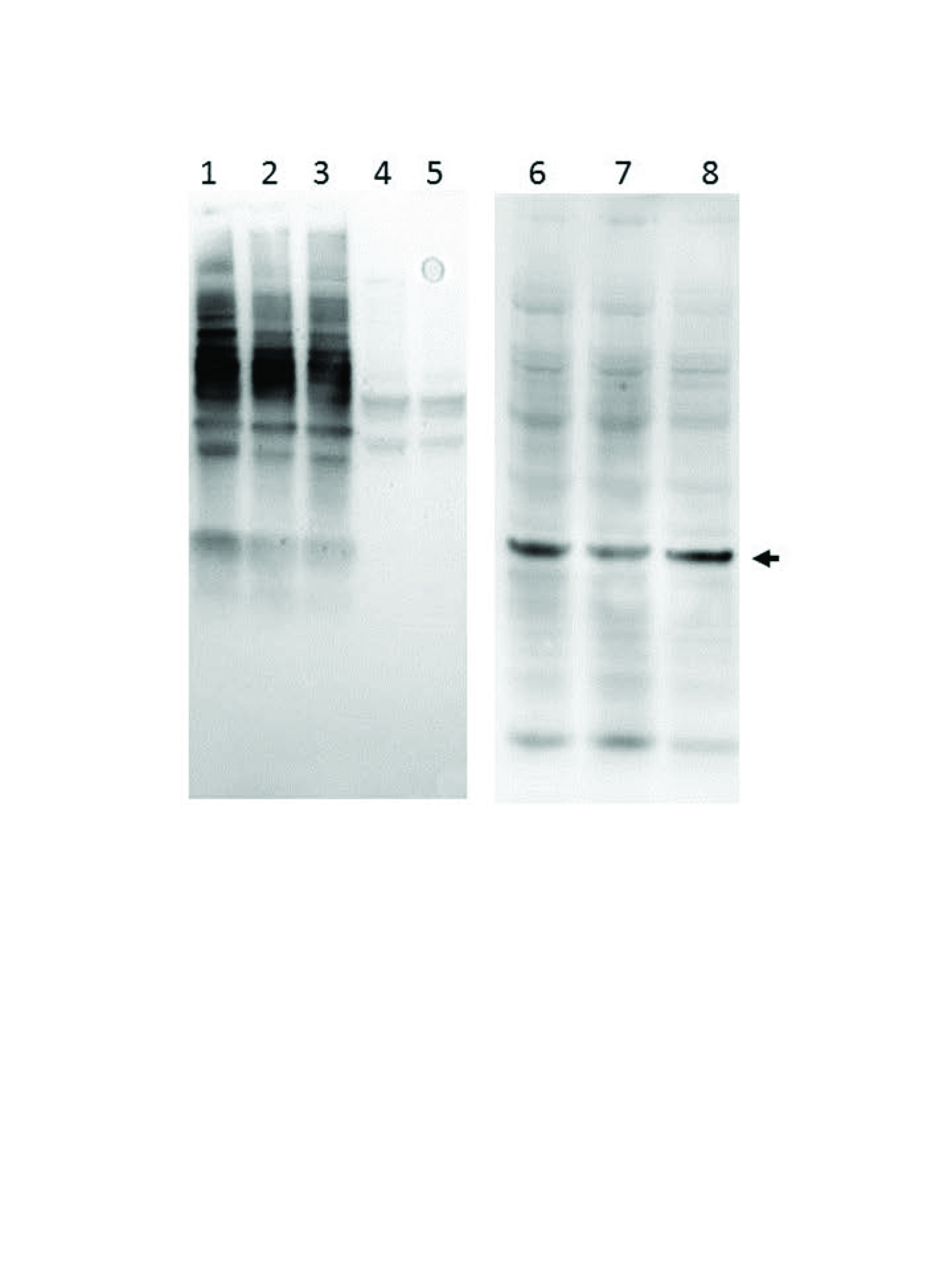

Supplement: S3 Fig — Tail skin samples from mice of BALB/c (lane 1), C57Bl/6 (lanes 2–3), and flaky tail (lanes 4–5) were homogenized in sample buffer. HEK-001 cells that were mock-transfected (lane 6), or transfected with filaggrin-directed siRNA (lane 7) or scrambled control siRNA (lane 8) for 48 hours were collected and extracted in whole cell lysis buffer. Samples were analyzed by SDS-PAGE and immunoblot using anti-filaggrin polyclonal antibody generated in rabbit. Arrow: 28 kDa, the predicted size of monomeric filaggrin. (TIF) [file pone.0170070.s003.tif]

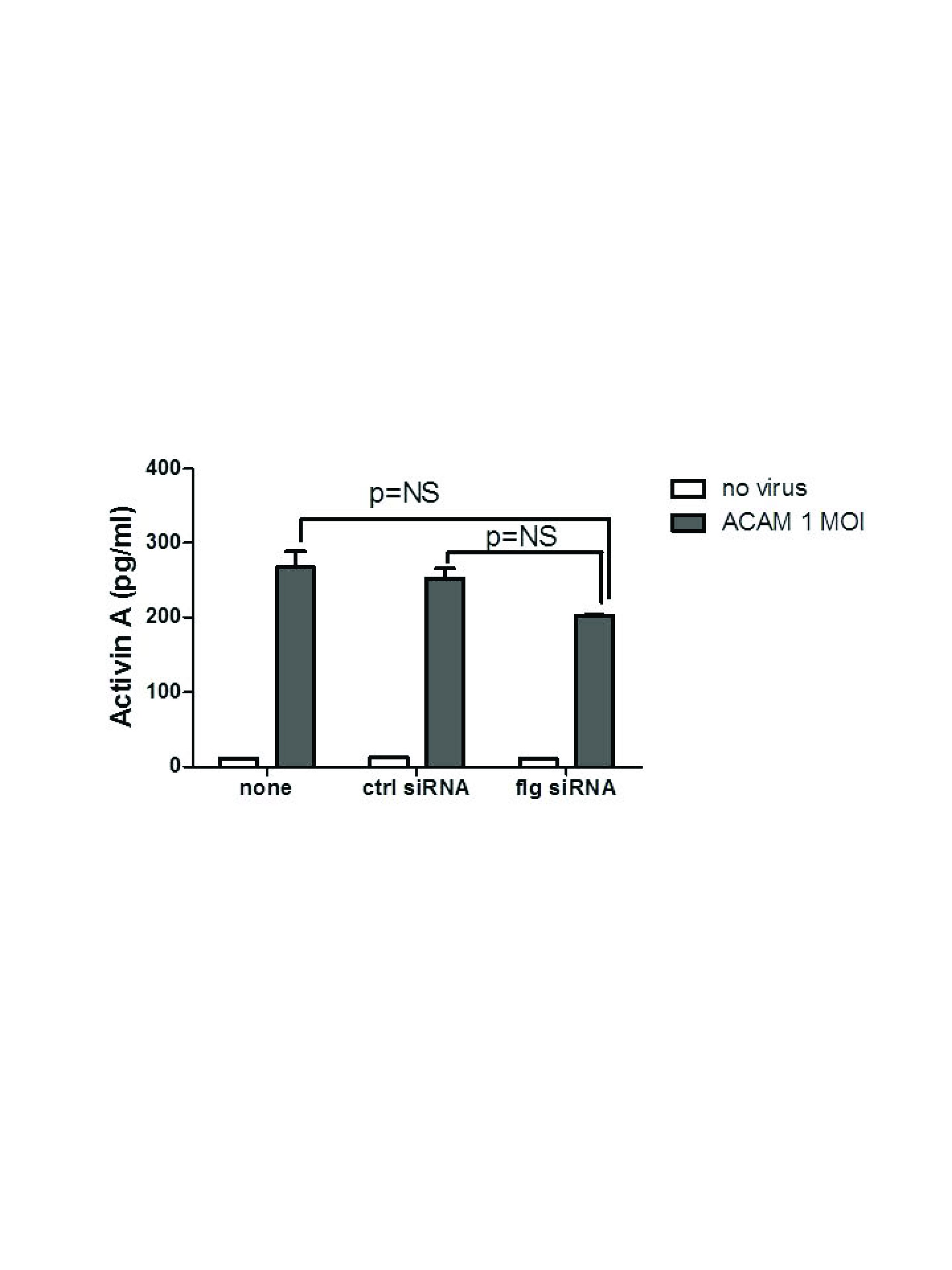

Supplement: S4 Fig — HEK-001 were mock-infected (white bar) or infected with ACAM-2000 at 20 MOI (grey bar). Activin A protein was assessed in supernatants collected at 48 hours post infection using a commercially available monoplex ELISA kit (n = 4). Small differences between groups were not statistically significant. (TIF) [file pone.0170070.s004.tif]

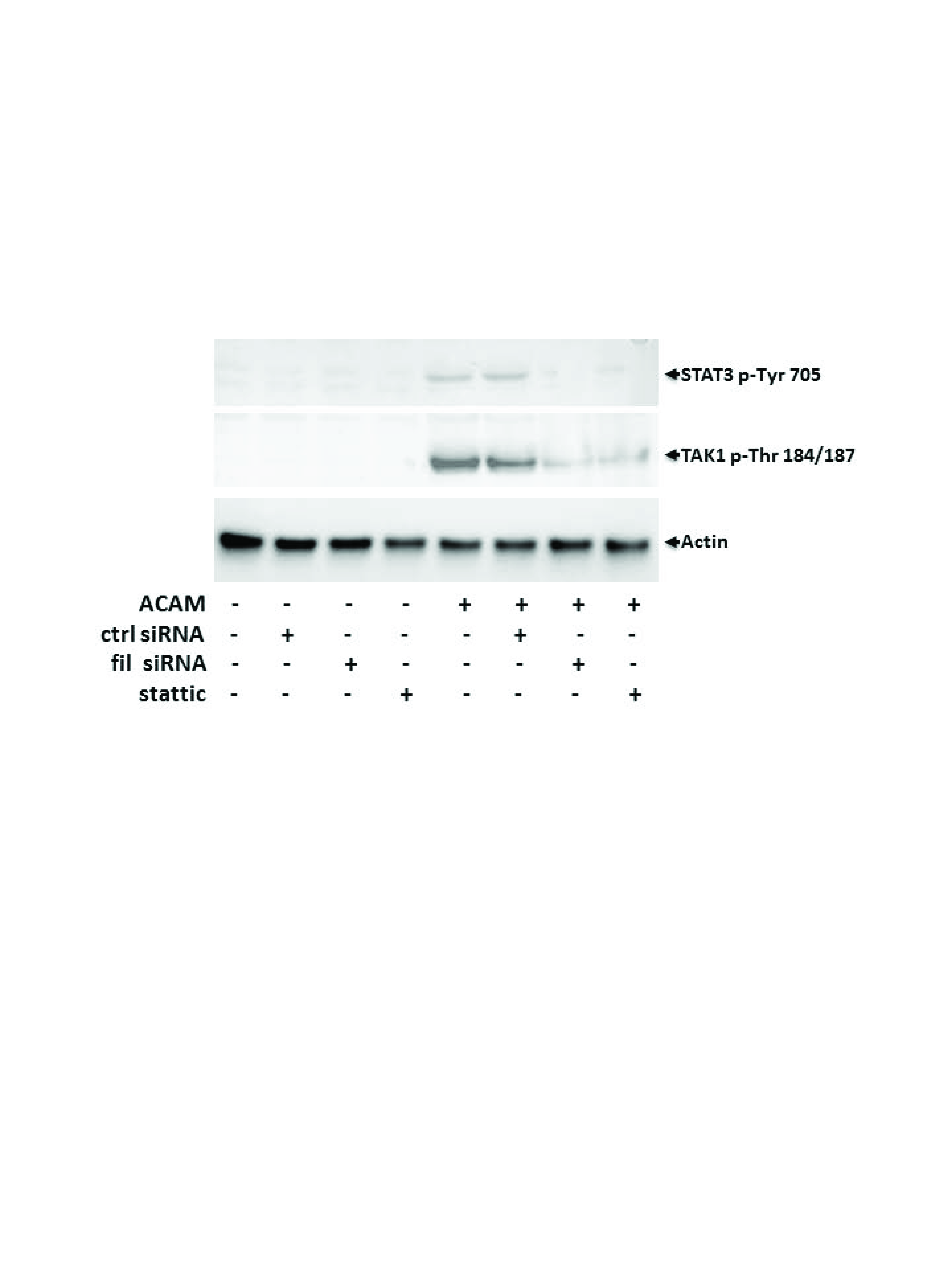

Supplement: S5 Fig — HEK-001 were infected with ACAM-2000 at 20 MOI. Hypotonic lysates collected at 3 hours post-infection were analyzed by immunoblot. Representative of 2 experiments is shown. (TIF) [file pone.0170070.s005.tif]
